# The cognitive and motor determinants of the perception of effort

**DOI:** 10.3758/s13415-025-01346-5

**Published:** 2025-10-01

**Authors:** Ela Herzberg, Israel Halperin, Noham Wolpe

**Affiliations:** 1 Department of Physical Therapy, The Stanley Steyer School of Health Professions, Gray Faculty of Medical & Health Sciences, Tel Aviv University, 6997801 Tel Aviv, Israel; 2 Sagol School of Neuroscience, Tel Aviv University, 6997801 Tel Aviv, Israel; 3 Department of Health Promotion, School of Public Health, Faculty of Medical & Health Sciences, Tel-Aviv University, Tel-Aviv, Israel; 4 Sylvan Adams Sports Institute, Tel Aviv University, Tel-Aviv, Israel

**Keywords:** Effort, Perception of effort, Depressive symptoms, Motivation

## Abstract

Effort refers to the physical work or force exerted to achieve an outcome, which is dissociable from the subjective experience that accompanies this exertion, termed perceived effort. Previous decision-making studies have examined effort valuation, focussing on individual differences in effort and reward sensitivity when choosing an action. These studies measure anticipatory aspects effort and reward, rather than their experiential aspects. Yet, how individuals perceive effort has significant implications for mental health. Here, we address this gap using an effort psychophysics task in young, healthy adults (*n* = 76). Participants used a hand dynamometer to raise a visual “mercury” column to a target zone, aiming to match the required force for at least 3 s within a 7-s window to succeed. After each trial, participants rated their perceived effort on a 0–100 visual analogue scale. We estimated the contribution of force and task failure to perceived effort ratings using a robust regression model. Higher depressive symptoms were associated with a reduced influence of exerted force and an increased influence of task outcome (failure) on perceived effort. We identified additional key cognitive and motor contributors to the experience of effort, such as accumulated fatigue, expectations and force stability. The experience of effort thus arises from multiple interacting cognitive and motor contributors. Individual differences in the contribution of these factors to the experience of effort, such as force and failure are associated with depressive symptoms, underscoring the importance of considering experiential aspects of effort in mental health research.

## Introduction

Effort can be defined in multiple ways. For instance, some researchers describe it as the work required to achieve a desired outcome (Wolpe et al., 2024), whereas others frame it as the energy expended to perform an action (Halperin & Vigotsky, 2024). Despite these differing definitions, there is a consensus that effort represents an objective process—distinct from the subjective experience associated with it: the perception of effort. The perception of effort varies considerably between individuals and has important implications for mental health and well-being. However, to date, much of the neuroscience research on effort has focused on effort valuation within decision-making paradigms (Berwian et al., 2020; Bonnelle et al., 2015; Le Heron et al., 2018; Treadway et al., 2009; Valton et al., 2024).

Studies on effort valuation typically examine how individuals make choices between two or more options differing in their effort requirements and associated rewards (Ang et al., 2023; Chong et al., 2017; Culbreth et al., 2018; Husain & Roiser, 2018; Müller et al., 2021). By using computational modelling, these paradigms allow researchers to quantify how much individuals are influenced by or sensitive to effort and reward when valuing different actions. This approach has yielded important insights into the computational and neural mechanisms underlying effort-based decision-making and its alterations in clinical populations. For example, individuals with depression often display reduced willingness to exert effort for rewards, suggesting an increased sensitivity to effort costs or decreased sensitivity to rewards during the decision-making process (Berwian et al., 2020; Treadway et al., 2012; but see Valton et al., 2024).

However, decision-making tasks primarily capture anticipatory aspects of effort and reward processing: how individuals value potential effort expenditure before actually experiencing it. They do not necessarily reflect the perception of effort during or immediately after task execution (Wolpe et al., 2024). Importantly, the experiential dimension of effort perception—how effortful an action feels—may not fully track actual effort expenditure (Halperin & Vigotsky, 2024). Indeed, there is evidence to suggest that the anticipation of effort during decision-making and the subjective experience of effort during action execution may be dissociable processes, both behaviourally (Xiao & Wolpe, 2025) and neurally (Kurniawan et al., 2013). This distinction between anticipatory and experiential aspects of effort processing is crucial, particularly when investigating motivational impairments in clinical conditions, which may show differential deficits in these processes (Wolpe et al., 2024). Moreover, effort-based decision-making tasks are typically designed to minimise failure rates, since failed trials introduce confounds in interpreting effort-reward trade-offs.

In the present study, we address these gaps by employing an effort psychophysics task that focuses on the perception of effort during physical exertion. First, unlike traditional decision-making paradigms, we focussed on how individuals perceive effort on a trial-by-trial basis as they produced varying levels of force in an isometric force matching task. Using the coefficients (slopes) from robust regression analyses, we computed individual differences in the contribution of force and outcome (i.e., whether participants failed in applying the required force for the required duration) when rating their perceived effort. Second, we investigated whether individual differences in these measures correlate with depressive and apathy symptoms, measured using the Patient Health Questionnaire-9 (PHQ9, Kroenke et al., 2001) and the Apathy Evaluation Scale (AES, Marin et al., 1991), respectively. We predicted that similar to the findings in the decision-making literature (see above), individuals with more severe depressive and apathy symptoms would show increased sensitivity to force and to failure when rating their effort experience. Finally, we explored additional trial-by-trial contributors to their perception of effort by computing several key cognitive and motor features and tested which features consistently influence effort rating across participants and across trials. These features, such as cumulative motor fatigue or force stability, are often neglected in decision-making studies (but see Müller et al., 2021). Thus using this approach, we sought to provide a more comprehensive account of the cognitive and motor determinants of the experience of effort.

## Methods

### Participants

We recruited participants through public ads at the university and social media platforms. Screening criteria included age of 18–45 years, no chronic medical condition, and no acute mental health condition requiring medication. We note that this criterion effectively excluded individuals with a current major depressive episode, owing to the typical use of antidepressant medication in such cases. Participants also had to pass a colour-blind test. All participants signed an informed consent prior to the start of the experiment. The study was approved by the Tel Aviv University Research Ethics Committee (reference 0005906). The experimental session took 40 min, and participants were remunerated 50 shekels for their time.

The principal analyses for which the study was powered for were correlations between the regression coefficients (slopes) for force and failure feedback and the two questionnaires (see below). Based on previous studies (Valton et al., 2024), a correlation of *r* = 0.3 would require *n* = 112 people for > 90% power to detect significant associations at ɑ = 0.05. We recruited 108 participants (62 females, 98 right-handed, mean age = 27.7 years, standard deviation [*SD*] = 5.8 years).

### Experimental procedure

**Fig. 1:**
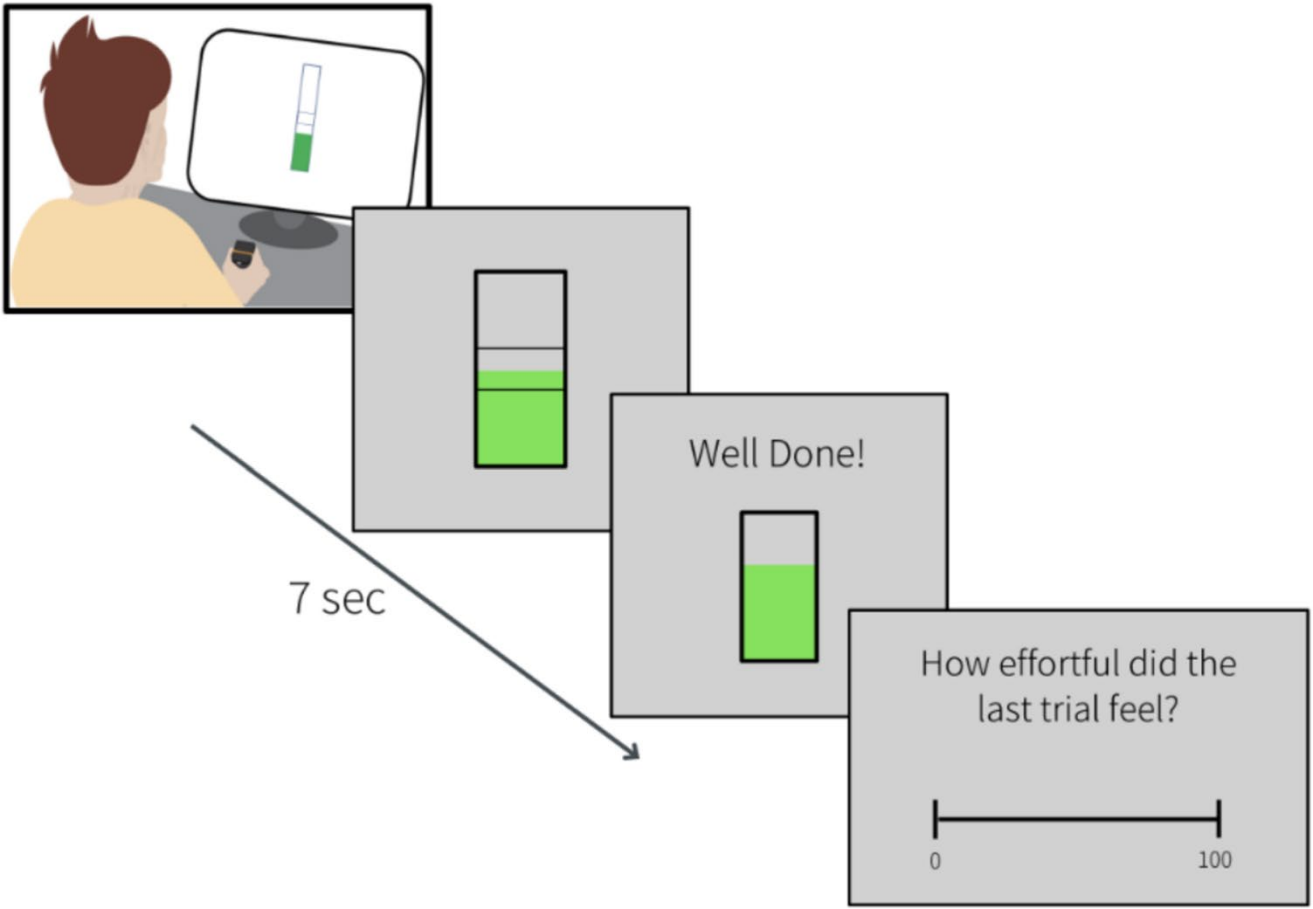
Task overview. Participants were asked to exert a target force using a hand dynamometer to make the green bar rise within the “thermometer” and stay in the target area for 3 s within a 7-s time window. The target force varied in relation to their Maximal Voluntary Contraction, and visual feedback was provided after each trial to indicate success or that the time was over. At the end of each trial, participants were asked to rate their level of perceived effort from 0 to 100

Participants completed a computerised physical effort task programmed in Python 3.8 using the PsychoPy library (Peirce et al., 2019). Overall, the task involved squeezing two hand dynamometers in order to reach a target area, followed by the rating of the subjective perception of effort (Fig. 1). Two hand dynamometers were used, one for each hand, and participants alternated between them on each trial to minimise muscle fatigue. Participants sat at a desk in front of a computer monitor and held the two dynamometers. Force was recorded using BIOPAC hand dynamometers (MP160) with a sample rate of 250 Hz.

After a brief familiarisation with the dynamometer and study setup, participants started the task with the measurement of their Maximal Voluntary Contraction (MVC). Participants were asked to squeeze the hand dynamometers as hard as they could for 3 s. Three repetitions were completed for each hand, and the maximum force was selected as each participant’s MVC, in line with standard procedures (Kurniawan et al., 2010).

In the main task, participants used a hand dynamometer to raise a visual “mercury” column into a target zone. Each trial lasted up to 7 s, during which participants were required to match the target force for a total of 3 s for the trial to be considered successful. Participants alternated between both hands for the task (dominant and nondominant) and the order of the trials (dominant or non-dominant first) was randomised across participants. Moreover, for clarity, each hand was associated with a different colour, which was used for the text font and mercury colour, and this association was also randomised across participants.

We adopted a “force-matching” design, requiring participants to maintain their exerted force within a specified target area, rather than exceeding a target force threshold, as done in many previous physical effort studies (Chong et al., 2017; Le Heron et al., 2018; Müller et al., 2021). The reasons for this were twofold: first, the force matching design meant that participants needed to exert a specific force magnitude, as we wanted to minimise the contribution of individual differences in exceeding a specific target force. This approach fits well with our study aim to test for the contribution of force magnitude to the perception of effort. Second, force matching makes the task more challenging by involving motor precision, as, unlike decision-making task, we wanted participants to have a proportion of failed trials to examine the contribution of failure to perceived effort. Each trial's target force magnitude (centre of the target area) was pseudo-randomly selected from a set of five forces {20%, 40%, 60%, 80%, 100%} relative to the participant’s MVC. The total area for each force level was ± 5% of the force level (e.g., 19–21% MVC, 38–42% MVC, etc.). The inclusion of 100% MVC was again aimed to increase failure rates for testing people’s sensitivity to success/failure. All target forces were pseudo-randomly repeated four times by each hand, except for the 100% MVC, which was repeated three times in an attempt to reduce fatigue, and the experiment was thus made up a total of 38 trials.

The target area remained visually constant for all trials and was fixed in the centre of the thermometer, so participants did not know the magnitude of the required force in a trial before squeezing the dynamometer. This was done to avoid potential biases in rating. After maintaining the force within the target area for the required duration or after time was up, participants received visual feedback indicating success or failure in each trial. After the feedback, participants were asked to rate their subjective effort perception on the last trial on a 0–100 visual-analogue scale using the computer mouse. Anchors were given as 0 for experiencing no effort at all (“no effort”) and 100 for experiencing the same amount of effort perceived during the MVC measurement (“maximal effort”). Once they confirmed their rating, the trial was completed, and a 20-s break was given before the subsequent trial started with the other hand.

Prior to the experimental task, participants underwent a colour blindness test as the stimuli were colour-coded (see above) and completed two questionnaires. The questionnaires included the AES (Marin et al., 1991) and the Patient Health Questionnaire 9 (PHQ9, Kroenke et al., 2001). These questionnaires were selected to assess depressive symptoms (PHQ9) and apathy (AES), as these constructs have been shown to be related to alterations in effort and reward sensitivity in clinical populations (Le Heron et al., 2018; Treadway et al., 2012) and in nonclinical individuals (Bustamante et al., 2023; Jurgelis et al., 2021; Norbury et al., 2024).

### Data analysis

We discovered a technical error in the task implementation, whereby target forces were incorrectly scaled according to the opposite hand’s MVC rather than the corresponding hand. This meant that for participants with large inter-hand MVC differences (typically with higher MVC in the dominant hand) the nondominant hand was presented with force targets that could exceed its own 100% MVC, whereas the dominant hand received lower-than-intended force levels. To account for this error in our analyses, we first recalculated the actual target force values used for each participant: for the dominant hand, forces were corrected using the formula (nondominant MVC/dominant MVC) × force_uncorrected; for the non-dominant hand, the formula was (dominant MVC/nondominant MVC) × force_uncorrected. This allowed us to accurately quantify the forces experienced by participants on each trial.

While this ensured we used the correct force levels for our analyses, participants were still presented with non-matching forces, both within participants across hands, and across participants. To minimise the impact of this issue, we excluded participants whose MVC ratio (dominant_MVC/(non-dominant_MVC) fell within the top quartile, resulting in a final sample of 81 participants. Moreover, we conducted sensitivity analyses restricted to participants with near-equal MVCs (ratio between 0.9 and 1.1, *n* = 26), for whom force targets were well matched across hands. As shown in the *Results*, our key findings were robust across different exclusion thresholds. Lastly, we included hand as a fixed-effect covariate in all regression models to control for any remaining hand-related variance in effort ratings.

Across participants, two exclusion criteria were applied to ensure data quality. First, we assumed participants who understood the task and engaged with it correctly would show a positive monotonic relationship between force and effort rating (before formally testing the relationship between force and effort rating, below). We therefore excluded participants with outlier (± 2.5 *SD* from mean) Spearman coefficient values for the correlation between force and effort rating (*M* = 0.813, *SD* = 0.146; one participant excluded). Second, we excluded participants with outlier values (± 2.5 *SD* from mean) of proportion of failed trials (*M* = 66.34%, *SD* = 23.34%; three participants excluded). We also excluded one participant who succeeded in all trials, as all regression analyses included trial outcome as a predictor, and 100% success rate would lead to rank deficiency. In total, 76 participants were included in the analyses.

We first examined the relationship between perceived effort and the force exerted using linear mixed-effects models. This analysis aimed to determine which function best described the relationship between target force and subjective effort rating. We fit four different linear mixed-effects models to the data using the following force transformation functions: linear (identity function), quadratic (parabolic), hyperbolic, and exponential functions, based on previous decision-making models (Chong et al., 2017). The best fitting model was selected using Bayesian Information Criteria (BIC) (Schwarz, 1978). The best fitting function was used in subsequent analyses.

Our principal analyses focussed on how individual differences in sensitivity to force and outcome when rating perceived effort were associated with depressive and apathy symptoms. To this end, we computed each participant’s sensitivity to force and outcome (failure) as the regression coefficients of force and failure predicting effort rating in a robust regression model. Hand was included as a covariate of no interest (see above). We then conducted two multiple regression analyses, with force sensitivity and failure sensitivity as the dependent variables, and PHQ9, AES, sex, age and MVC ratio (to control for residual hand difference effect, see above) the independent variables. All continuous variables were z-scored before entered to the regression model. False discovery rate (FDR) correction was used to correct for four comparisons (PHQ9 and AES predicting force and failure sensitivity).

To complement the association of force and outcome sensitivity with affective symptoms, we explored which additional cognitive and sensorimotor factors consistently influence trial-by-trial effort rating. The features, their presumed sensorimotor/cognitive interpretation and their calculation are summarised in Table 1. For computing the initial force difference, we computed the difference between the first peak of force and the target force, where the first peak was identified using the *signal.find_peaks* function from the Scipy library (Virtanen et al., 2020), with a required minimal height of 0.1. The sum of force exerted in the experiment was calculated as the cumulative area under the force curve until the current trial, including the current trial.

**Table 1:** Summary of all extracted features for the analysis predicting effort rating across trials

Feature	Cognitive/motor factor	Calculation
Target force	Goal	Target force required in a trial
Failure	Negative outcome	Determined according to task performance
Effort rating (t-1)	Perseveration	From the previous trial
Target force (t-1)	Relative expectation	From the previous trial
Sum of force exerted	Cumulative motor fatigue	Cumulative sum of area under the force curve across trials
Coefficient of variation	Force stability	Force standard deviation over force mean throughout the trial
Initial force difference	Initial overshoot	Initial force peak minus target force
Excess force	Additional force exerted on top of that required in the trial	Area under the curve of actual force trace minus target force times the time required to maintain force in the trial

Excess force exerted on a given trial was calculated as the difference between the actual area under the force curve and the ideal area required for success. The ideal area was defined as the product of the target force and the duration needed to meet the success criterion (i.e., 3 s).

We explored the consistency of these features in predicting the subjective effort ratings. To account for outliers, heteroscedasticity in subjective ratings, and high collinearity between some predictors, we used a two-step approach. First, we fit a robust linear model using Huber’s M-estimator to derive trial-level weights. These weights were then passed to a cross-validated ridge regression model to estimate regularised beta coefficients. Ridge regression was chosen specifically because it is well-suited to high multicollinearity, as it shrinks the coefficients of correlated predictors while retaining them in the model, thereby allowing more stable estimation of their relative contributions. The optimal shrinkage parameter (lambda) was selected from tenfold cross-validation based on the minimum mean squared error. Final beta estimates for each predictor were extracted for each participant and summarised across the group.

All analyses were performed with Matlab (Mathworks, MA) and Python (version 3.10) using NumPy (Harris et al., 2020), Pandas (The pandas development team, 2024), Scikit (Pedregosa et al., 2011), and Scipy (Virtanen et al., 2020) libraries. Figures were generated in R Version 4.4.1 (R Core Team, 2016) with ggplot2 (Wickham, 2016).

## Results

A summary of demographics and questionnaire scores for participants included in the analyses are summarised in Table 2.

**Table 2:** Demographics and questionnaire scores for participants included in the analyses

Variable	Summary
No. participants	76
Age (mean ± SD)	27.07 ± 5.15
Sex (female/male)	43/33
Dominant hand (right/left)	67/6
PHQ9 score (mean ± SD)	6.41 ± $4.03$
AES score (mean ± SD)	31.41 ± 6.82

PHQ9 = Patient Health Questoinnaire-9; AES = Apathy Evaluation Scale

### Relationship between effort rating and force

**Fig. 2:**
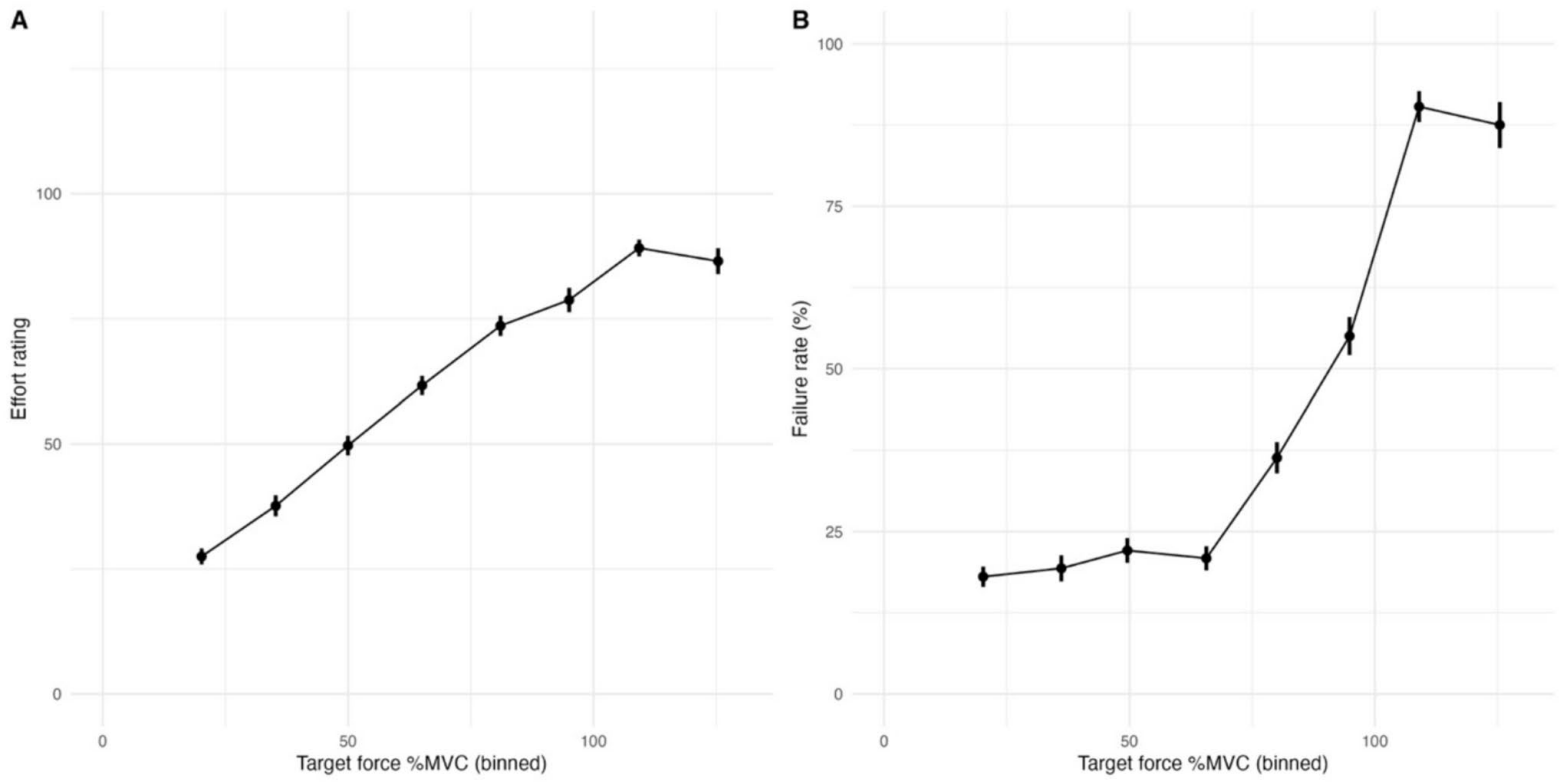
Effort rating and failure rate as a function of target force across participants. **A**) Mean subjective effort rating as a function of binned target force. Target force values (expressed as a percentage of maximum voluntary contraction, %MVC) were divided into equal-width bins. For each participant, mean effort ratings were calculated within each bin and then averaged across participants to compute the grand mean. Error bars represent the standard error of the mean (SEM) across participants. For illustration and SEM calculation, only bins with data from more than one participant are shown. **B**) Same as (A), but for failure rate as a function of binned target force

Overall, participants consistently increased their rating with increasing target force (Fig. 2A). Moreover, participants’ failure rate increased with increasing target force, as expected (Fig. 2B). We first assessed which function best described the relationship between the force exerted and effort rating in order to implement it in subsequent analyses. To this end, we fit four different mixed-effects models predicting effort rating from force, with effort transformed using one of the four functions: linear (identity function), quadratic, hyperbolic and exponential (Chong et al., 2017). Model goodness-of-fit (BIC) values are shown in Table 3.

**Table 3:** Function linking effort rating and target force

	Bayesian information criterion	
Link function	Random intercept	Random intercept and random slope
Linear	− 2,574	− 2,907
Quadratic	− 2,948	− 2,984*
Hyperbolic	− 1,707	− 1,769
Exponential	− 2,150	− 2,390

Goodness-of-fit measures (Bayesian Information Criterion) for the four models predicting effort rating using the four link functions. Hand was included as a fixed effect. *Best fit

The best fitting model was the model with a quadratic function which had random intercept and slope (random slope suggests importance for individual differences, as we expected in this study). A quadratic function is consistent with decision-making studies which is often found to best describe the relationship between force and subjective value in decision-making studies (Chong et al., 2017) and with studies involving explicit effort rating (Weilharter et al., 2024). We therefore used the quadratic function in all subsequent analyses.

### Association between force sensitivity and failure sensitivity with depressive symptoms

We next tested for associations between individual differences in the contribution of force and failure to effort rating and depressive and apathy symptoms, measured with PHQ9 and AES, respectively. To compute force and failure sensitivity in our task, we fit a robust linear regression model to each participant’s effort rating behaviour, with force (quadratic function) and outcome feedback (coded as 1 and 0 for failure and success, respectively) as predictors. These measures of force sensitivity and failure sensitivity were entered as the dependent variable in two regression analyses, with PHQ9 and AES as the main predictors of interest, and age, sex and MVC ratio as covariates of no interest (see *Methods*).

**Fig. 3:**
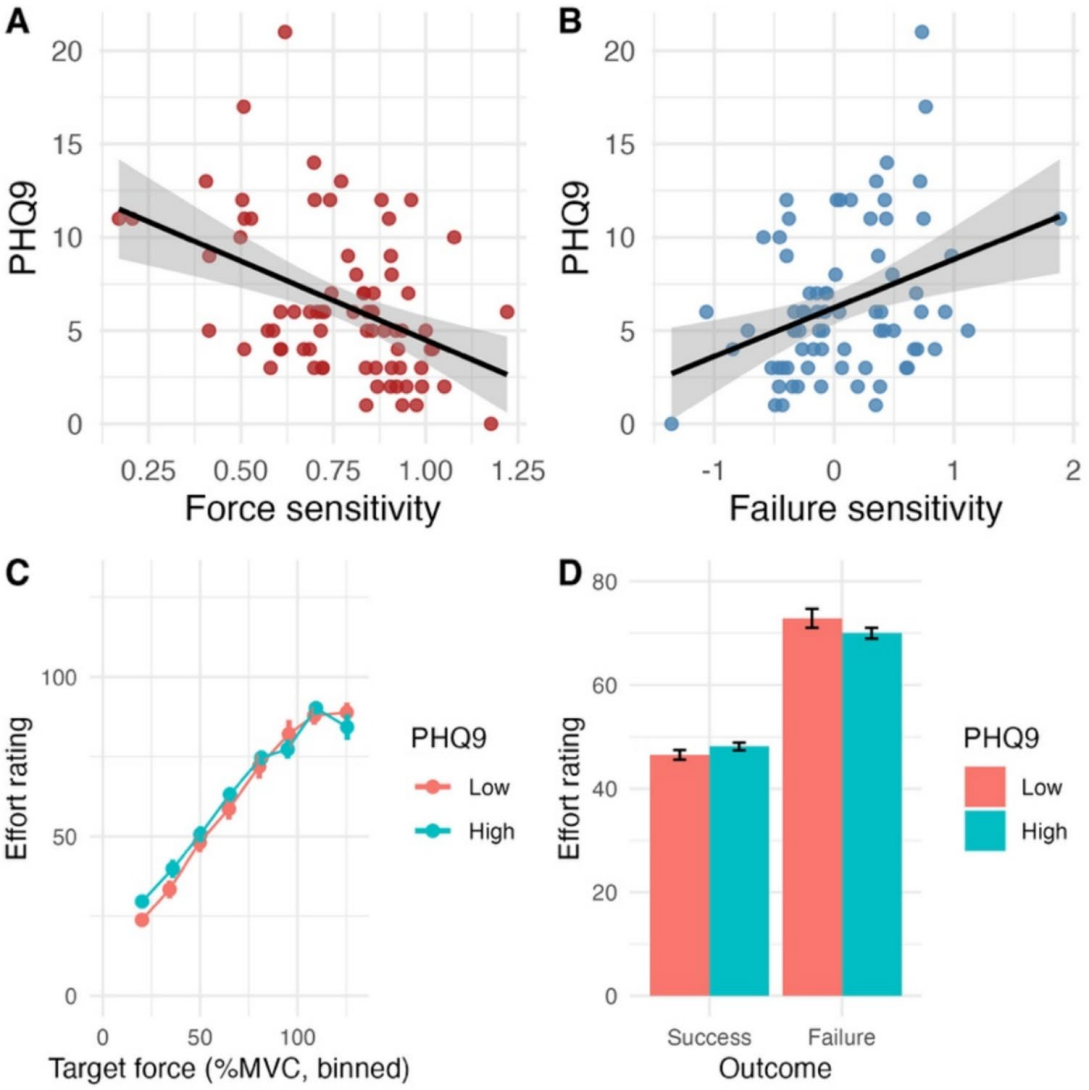
Force and failure sensitivity across depressive symptoms. **A**) Scatter plot showing the association between depressive symptom severity, measured using Patient Health Questionnaire-9 (PHQ9) and force sensitivity computed from the robust regression model for each participant. Black line and shaded grey area indicate linear fit and 95% confidence interval, respectively. We note that removing the two participants with high PHQ9 values did not change the results. **B**) Same as (**A**) but for the association between PHQ9 and failure sensitivity computed from the robust regression across participants. We note that removing the two participants with high PHQ9 values did not change the results. **C**) Mean (and SEM) of subjective effort rating as a function of binned target force (as in Fig. 2A), plotted separately for individuals with low (orange) and high (cyan) PHQ9 scores. Low and high PHQ9 scores were determined using a median split (median = 6, *n* = 37 and 38 individuals per group, respectively). Note that the group separation was done for illustration purposes only, and all analyses were conducted with PHQ9 as a continuous variable. **D**) As in (**C**), but for mean (and SEM) of subjective rating as a function of trial outcome (success vs. failure) for individuals with low (orange) and high (cyan) PHQ9 scores

We found significant FDR-corrected associations between force sensitivity and failure sensitivity with depressive symptoms. Specifically, there was negative association between force sensitivity and depressive symptoms (*β* =  − 0.467, *p* = 0.002), but not between force sensitivity and apathy symptoms (*β* = 0.017, *p* = 0.903). Moreover, there was a significant positive association between failure sensitivity and depressive symptoms (*β* =  − 0.455, *p* = 0.004) but not between failure sensitivity and apathy symptoms (*β* = 0.147, *p* = 0.44) (Fig. 3). A sensitivity analysis, including only participants with force range 18–110% MVC (*n* = 26), showed similar associations between depressive symptoms and force sensitivity (*β* =  − 0.392) and depressive symptoms and failure sensitivity (*β* = 0.344). Similarly, examining sensitivity to the actual force exerted (computed as the area under the force curve) instead of target force produced a similar association with depressive symptoms (*β* =  − 0.35,* p* = 0.01*,* FDR-corrected).

As we predicted a positive association between depressive symptoms and apathy and sensitivity to force in effort ratings, we examined whether the observed negative association could be explained by a compression or ceiling effect—specifically, that individuals with higher depressive symptoms might exhibit a shallower force–perceived effort relationship, because they anchor their effort rating higher and/or have more frequent use of the maximum rating. We found support for the former but not the latter: there was a positive correlation between depressive symptoms and the intercept (*r* = 0.325, *p* = 0.005, uncorrected); removing trials with a rating of > 90 did not alter these results (*r* =  − 0.2411, *p* = 0.037, uncorrected). Together, the results suggest that individuals with more depressive symptoms tend to be less influenced by force magnitude and more influenced by failure feedback when experiencing effort. Although higher depressive symptoms were associated with elevated overall effort ratings, the use of multiple regression isolates the unique contributions of force and failure sensitivity, independent of this intercept shift. We next explored the unique contributions of other cognitive and motor features to trial-by-trial effort rating across participants.

### Additional cognitive and motor determinants of effort rating

We next explored additional cognitive and motor determinants influencing trial-by-trial effort perception across participants. We first extracted several features which we predicted would influence effort rating, over and above target force and failure feedback. The trial-level features included: 1) effort rating in the previous trial, reflecting perseveration; 2) target force in the previous trial, reflecting relative expectation; 3) cumulative sum of force exerted by the participant in the experiment, reflecting fatigue. Moreover, we computed the following within-trial features from each trial’s force trace: 1) the Coefficient of Variation, reflecting force instability (variability in force production); 2) initial force overshoot or undershoot relative to the target, reflecting anticipatory components; and 3) excess force, reflecting the additional force exerted over time in the trial on top of the required force.

**Fig. 4:**
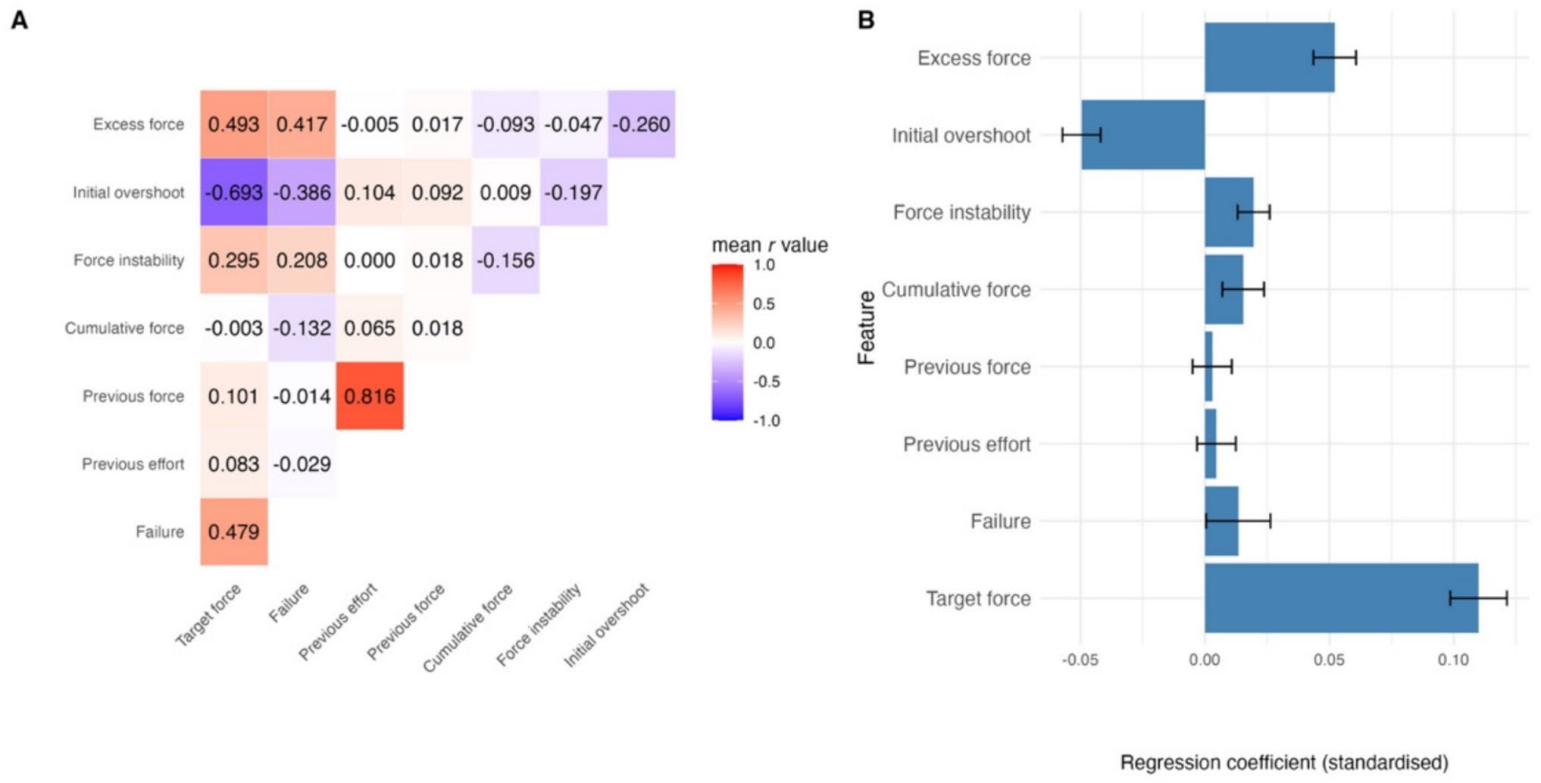
Cognitive and motor features predicting effort rating. **A**) Heatmap showing the mean Pearson correlation coefficients across participants between the extracted features hypothesised to influence trial-by-trial effort rating. Warm colours represent positive correlations, and cool colours represent negative correlations. **B**) Standardised regression coefficients for each of the extract features from the robust ridge regression conducted for each participant. Ridge regularisation was applied to account for collinearity between predictors. Hand was included in the model as a regressor of no interest. Error bars indicate 95% confidence interval

The extracted features were differentially associated with one another (Fig. 4A). Given their collinearity, we employed a ridge-regularised robust regression approach, which is designed to handle shared variance among predictors by shrinking coefficients while retaining all features. This enabled us to estimate each participant’s unique weighting of each feature despite covariance. We found that some features showed high consistency across participants while others showed high individual variability in their contribution to effort rating (Fig. 4B). Specifically, as expected, target force had the highest positive effect on effort rating across participants. Similarly, excess force (force exerted on top of that required in the trial), force instability and cumulative force (fatigue) all had a relatively consistent positive effect on effort rating. Failure feedback, conversely, showed a positive effect on effort rating, but high individual variability was observed (see also Fig. 2B, abscissa). Lastly, initial force overshoot had a consistent and relatively large negative effect on effort rating, such that high initial overshoot predicted a lower effort rating at the end of the trial, and vice versa.

## Discussion

Our study had three aims: 1) to quantify individual differences in how effort, operationalised in our study as the force required to achieve a task, and failure attaining the goal, influenced the perception of effort; 2) to test for correlations with depressive and apathy symptoms; and 3) to explore additional cognitive and motor contributors to the perception of effort. We found large individual variability in how target force and failure influence the perception of effort. Individual differences in how these factors influence effort perception negatively correlated with depressive symptoms. We did not find any associations with apathy, although this may reflect low power (see limitations below). Lastly, we identified cognitive and motor features that consistently change effort rating.

Effort and reward sensitivity are commonly investigated in decision-making research (Berwian et al., 2020; Chong et al., 2017, 2018, 2023; Culbreth et al., 2018; Husain & Roiser, 2018), where they are typically computed as how much effort and monetary reward shifts the probability of choosing certain actions. However, decision-making studies focus on anticipatory aspects of effort valuation. Moreover, decision-making tasks are designed to maximise success rate once a decision is made, which makes it difficult to separate the effect of failure on future effort and reward valuation.

In contrast to decision-making studies, our primary analyses focussed on the experiential aspects of effort and outcome sensitivity. In our study, sensitivity refers to how these factors contribute to the perception of effort, which we computed as the individual robust regression coefficients (slopes) of these factors in predicting effort rating. Using a force matching design, we aimed to use the target force as a proxy for objective effort and to induce occasional failures in order to examine how task failure influences perceived effort. We note, however, that in the commonly used force threshold/exceedance design, the trial-level target force may not accurately reflect the effort actually exerted. In such cases, alternative measures, such as the area under the force curve, may serve as better proxies for effort, which could alter the results obtained with this alternative design.

We found a strong and consistent quadratic relationship between target force and perceived effort across participants, such that perceived effort increased with the square of target force. This finding aligns with previous studies that have examined the association between objective and subjective effort across various physical tasks, including the grip task used in the present study (Weilharter et al., 2024). It further supports the idea that, although the relationship between actual and perceived effort is not always linear, the two tend to track closely—at least within the context of physical exertion (Halperin & Vigotsky, 2024). Interestingly, a quadratic relationship is often found to best explain the relationship between objective effort and action value in decision-making studies (Chong et al., 2017). This convergence could be explained by optimal motor control theories, which suggest that the cost of exerting control (or effort) is proportional to the square of the control signal (Manohar et al., 2015). However, it remains to be seen whether this quadratic relationship also holds in force-threshold designs, beyond the force-matching design used in the present study.

How much force influences the trial-by-trial perception of effort correlated with depressive symptoms, such that individuals with more depressive symptoms showed a reduced tendency to be influenced by force magnitude when rating effort. Although our study examined individuals who were not clinically diagnosed with depression, adopting a dimensional psychiatry approach (Hägele et al., 2015), we expected that individuals with more depressive symptoms would be more sensitive and more influenced by force when rating their perceived effort. This hypothesis was based on decision-making literature in clinical (Berwian et al., 2020; Bustamante et al., 2024; Cléry-Melin et al., 2011; Vinckier et al., 2022; but see Valton et al., 2024) and nonclinical depression (Bustamante et al., 2023; Jurgelis et al., 2021; Norbury et al., 2024).

The negative association between force-rating coupling and depressive symptoms could be partly driven by a possible rating compression or anchoring effect, as individuals with higher depressive symptoms also showed higher intercept in rating. Importantly, however, as the associations between depressive symptoms and both force sensitivity and outcome sensitivity were estimated within the same multiple regression model, the observed relationships between depression and sensitivity to force or failure cannot be solely attributed to differences in intercept. Moreover, the effect was not driven by a rating ceiling effect, as removing trials with high ratings did not alter the negative relationship between force-rating coupling and depressive symptoms.

What other mechanisms could drive the negative association between depressive symptoms and experiential force-rating coupling? Individuals with clinical depression (Dunne et al., 2021) or subclinical depressive symptoms (Ruggiero et al., 2025) show reduced interoceptive ability, which is also reflected in reduced insular activity during interoceptive tasks (Avery et al., 2014). Perceptual insensitivity (Smith et al., 2021) and impaired interoceptive-exteroceptive integration have been proposed to be a key mechanism in depressive symptoms (Harshaw, 2015). Reduced interoception, coupled with a general negative bias in rating, could together lead to the high effort rating and shallower force-perceived effort slopes we found in association with depressive symptoms. Interestingly, a recent study found that individuals with schizophrenia with varying degrees of motivational and cognitive symptoms show a similar flattening of the force-effort rating curve in a force matching effort design compared with controls (Fig. 4B in Culbreth et al., 2024) but more sensitive to effort in a decision-making task. These results suggest the flattening of the force-perceived effort relationship may also be related to cognitive mechanisms related to motivation (Wolpe et al., 2025), and can be distinct from effort sensitivity during decision-making (Culbreth et al., 2024).

In contrast to the overall consistent relationship between force and perception of effort, there was a large individual variability in how failure feedback influences the perception of effort. Many individuals showed negative regression coefficients, suggesting that in these individuals, failure outcome was associated with lower effort rating. This variability could stem from individual differences in interpretation of feedback, or sensitivity to reward/punishment cues. For example, some individuals might interpret success as evidence that high effort was worthwhile or justified, thus retrospectively increasing their perceived experience. In contrast, others might associate success with ease or efficiency, thereby experiencing lower perceived effort following success. Exploring personality traits, such as attribution styles could further clarify why such different interpretations occur across individuals.

Explaining some of the variability in responses to feedback, we found that more depressed individuals tended to be more influenced by failure feedback. The positive association between failure sensitivity in effort rating and depressive symptom is consistent with the well-known finding that depressed individuals tend to be particularly sensitive to failure (Elliott et al., 1997). This result is further consistent with the psychological observation that depression is associated with increased sensitivity to negative outcomes but reduced sensitivity to positive outcomes (Rottenberg et al., 2005). In decision-making tasks, depressive symptoms are associated with a shift towards a loss minimisation strategy (Maddox et al., 2012).

While different from monetary reward typically used in decision-making tasks, the setup in our experiment was more naturalistic as participants were provided with veridical feedback about their performance and we examined how this feedback influenced their rating. As value-based decision-making models typically ignore performance measures, these tasks are typically designed for ceiling level performance (Xiao & Wolpe, 2025). Accounting for individual differences in outcome feedback on effort perception is a major strength of our setup.

Although our study focussed on force and failure sensitivity in the perception of effort, we explored the contribution of additional cognitive and motor features to perceived effort. We found a few features that consistently contributed to increasing perceived effort across participants, namely: cumulative force exerted in the experiment reflecting fatigue, force exerted on top of the target force and variation in force production. By contrast, initial force overshoot or undershoot reflecting prediction error contributed to reducing the perception of effort, such that if a participant exerted more force than needed at the start of the trial, they will likely rate the trial as less effortful, and vice versa. Our study was not specifically designed or sufficiently powered to examine correlations between these nuanced features and trait-level individual differences across various scales. However, future research could investigate whether specific mental health factors are associated with differential sensitivity to these experiential components of effort.

Although our study was conducted in an overall healthy population and requires validation in clinical population with depression, our results underscore the importance of studying fluctuations in the perception of effort—not just in anticipated effort valuation—as a potential core construct in psychopathology. Previous studies have primarily focused on anticipatory decision-making contexts, where individuals decide whether to engage in a task given its cost (effort) and benefit (reward). In contrast, our paradigm directly probed the subjective experience of effort after motor execution. This distinction is crucial as anticipatory and experiential effort are dissociable behaviourally (Wolpe et al., 2024) and neurally: anticipatory effort valuation has been linked to dopaminergic pathways, such as the ventral striatum in particular (Salamone et al., 2016), and their connections with the anterior cingulate cortex and the ventromedial prefrontal cortex which encodes the subjective value of actions (Hogan et al., 2019; Lopez-Gamundi et al., 2021). By contrast, experiential aspects of effort, such as perceived exertion, are supported by sensorimotor and insular networks (De Morree & Marcora, 2015; Hu et al., 2022). Our results support this distinction, and suggest that anticipatory and experiential aspects of effort can be differentially related to mental health factors (Wolpe et al., 2024). Future research can investigate whether and, if so, how anticipatory effort valuation and perception of effort relate to one another within the same individual (Xiao & Wolpe, 2025).

Our study has several strengths and limitations. A strength of this study lies in its use of a simple and intuitive psychophysics task that allows for quantification of cognitive and motor features for modelling subjective ratings across trials. The robust regression approach accounted for individual variability and potential outliers, and the use of ridge regression reduced overfitting while capturing the joint contribution of multiple predictors. However, several limitations should be acknowledged. First, the force stimuli were inadvertently miscalibrated across hands owing to a technical error in MVC normalisation. While this was addressed via exclusion and covariate control, it remains a potential confound in our study and resulted in the exclusion of many participants which lowered the expected power. Second, the use of target area in the task was meant to increase failure and match exerted and required force, however, it also added a precision element which is absent in many cognitive neuroscience tasks using hand dynamometers. Third, the study tested people from the general population, and the correlations with depressive symptoms should be validated in a clinical population. Fourth, we focussed on physical effort and did not measure cognitive effort in this study. This is largely because physical effort can be more easily operationalised and measured. While we believe our findings could be relevant to the cognitive domain, this remains to be validated. Fifth, acknowledge that sensitivity to action outcome is likely different to sensitivity to monetary reward, and future research using a similar setup can directly manipulate trial-by-trial reward to investigate how it influences effort perception.

## Conclusions

This study provides evidence subjective effort perception is shaped by effort and outcome sensitivity. Importantly, individual differences in how participants weighed these factors were associated with depressive symptoms but not apathy, underscoring the importance of individual differences in the factors contributing to the perception of effort. In addition, we found multiple interacting cognitive and motor features that contribute to effort perception, including motor prediction error, force variability, and fatigue. Together with prior neuroimaging work, our findings support a dissociation between neural systems involved in effort anticipation and those underpinning the moment-to-moment experience of effort. A more nuanced understanding of effort perception may help refine clinical models of motivational impairments and guide targeted interventions in affective disorders.

## Data Availability

All data used for the analyses in this study is available at https://github.com/nwolpe/effort_perception.

## Code availability

Code used to analyse the data and create the figures is available at https://github.com/nwolpe/effort_perception.

## Declarations

### Open Practices Statement

Data and the code used to analyse the data and create the figures are available at: https://github.com/nwolpe/effort_perception.

## References

[CR1] Ang, Y.-S., Gelda, S. E., & Pizzagalli, D. A. (2023). Cognitive effort-based decision-making in major depressive disorder. *Psychological Medicine,**53*(9), 4228–4235. 10.1017/S0033291722000964 35466895 10.1017/S0033291722000964

[CR2] Avery, J. A., Drevets, W. C., Moseman, S. E., Bodurka, J., Barcalow, J. C., & Simmons, W. K. (2014). Major depressive disorder is associated with abnormal interoceptive activity and functional connectivity in the insula. *Biological Psychiatry,**76*(3), 258–266. 10.1016/j.biopsych.2013.11.027 24387823 10.1016/j.biopsych.2013.11.027 PMC4048794

[CR3] Berwian, I. M., Wenzel, J. G., Collins, A. G. E., Seifritz, E., Stephan, K. E., Walter, H., & Huys, Q. J. M. (2020). Computational mechanisms of effort and reward decisions in patients with depression and their association with relapse after antidepressant discontinuation. *JAMA Psychiatry,**77*(5), 513. 10.1001/jamapsychiatry.2019.4971 32074255 10.1001/jamapsychiatry.2019.4971 PMC7042923

[CR4] Bonnelle, V., Veromann, K.-R., Heyes, S. B., Sterzo, E. L., Manohar, S., & Husain, M. (2015). Characterization of reward and effort mechanisms in apathy. *Journal of Physiology-Paris,**109*(1–3), 16–26. 24747776 10.1016/j.jphysparis.2014.04.002 PMC4451957

[CR5] Bustamante, L. A., Barch, D. M., Solis, J., Oshinowo, T., Grahek, I., Konova, A. B., ..., Cohen, J. D. (2024). Major depression symptom severity associations with willingness to exert effort and patch foraging strategy. *Psychological Medicine,**54*(15), 4396–4407. 10.1017/S0033291724002691 39618329 10.1017/S0033291724002691 PMC11650159

[CR6] Bustamante, L. A., Oshinowo, T., Lee, J. R., Tong, E., Burton, A. R., Shenhav, A., ..., Daw, N. D. (2023). Effort foraging task reveals positive correlation between individual differences in the cost of cognitive and physical effort in humans. *Proceedings of the National Academy of Sciences of the United States of America,**120*(50), Article e2221510120. 10.1073/pnas.2221510120 38064507 10.1073/pnas.2221510120 PMC10723129

[CR7] Chong, T.T.-J., Apps, M. A. J., Giehl, K., Hall, S., Clifton, C. H., & Husain, M. (2018). Computational modelling reveals distinct patterns of cognitive and physical motivation in elite athletes. *Scientific Reports,**8*(1), Article 11888. 10.1038/s41598-018-30220-3 30089782 10.1038/s41598-018-30220-3 PMC6082862

[CR8] Chong, T.T.-J., Apps, M., Giehl, K., Sillence, A., Grima, L. L., & Husain, M. (2017). Neurocomputational mechanisms underlying subjective valuation of effort costs. *PLoS Biology,**15*(2), Article e1002598. 10.1371/journal.pbio.1002598 28234892 10.1371/journal.pbio.1002598 PMC5325181

[CR9] Chong, T.T.-J., Fortunato, E., & Bellgrove, M. A. (2023). Amphetamines improve the motivation to invest effort in attention-deficit/hyperactivity disorder. *Journal of Neuroscience,**43*(41), 6898–6908. 10.1523/JNEUROSCI.0982-23.2023 37666665 10.1523/JNEUROSCI.0982-23.2023 PMC10573750

[CR10] Cléry-Melin, M.-L., Schmidt, L., Lafargue, G., Baup, N., Fossati, P., & Pessiglione, M. (2011). Why don’t you try harder? An investigation of effort production in major depression. *PLoS One,**6*(8), Article e23178. 10.1371/journal.pone.0023178 21853083 10.1371/journal.pone.0023178 PMC3154289

[CR11] Culbreth, A. J., Chib, V. S., Riaz, S. S., Manohar, S. G., Husain, M., Waltz, J. A., & Gold, J. M. (2024). Increased sensitivity to effort and perception of effort in people with schizophrenia. *Schizophrenia Bulletin*. 10.1093/schbul/sbae162 39312272 10.1093/schbul/sbae162 PMC12061651

[CR12] Culbreth, A. J., Moran, E. K., & Barch, D. M. (2018). Effort-based decision-making in schizophrenia. *Current Opinion in Behavioral Sciences,**22*, 1–6. 10.1016/j.cobeha.2017.12.003 29607387 10.1016/j.cobeha.2017.12.003 PMC5875438

[CR13] De Morree, H. M., & Marcora, S. M. (2015). Psychobiology of Perceived Effort During Physical Tasks. In G. H. E. Gendolla, M. Tops, & S. L. Koole (Eds.), *Handbook of Biobehavioral Approaches to Self-Regulation* (pp. 255–270). Springer New York. 10.1007/978-1-4939-1236-0_17

[CR14] Dunne, J., Flores, M., Gawande, R., & Schuman-Olivier, Z. (2021). Losing trust in body sensations: Interoceptive awareness and depression symptom severity among primary care patients. *Journal Of Affective Disorders,**282*, 1210–1219. 10.1016/j.jad.2020.12.092 33601698 10.1016/j.jad.2020.12.092 PMC10398840

[CR15] Elliott, R., Sahakian, B. J., Herrod, J. J., Robbins, T. W., & Paykel, E. S. (1997). Abnormal response to negative feedback in unipolar depression: Evidence for a diagnosis specific impairment. *Journal of Neurology, Neurosurgery & Psychiatry,**63*(1), 74–82. 10.1136/jnnp.63.1.74 9221971 10.1136/jnnp.63.1.74 PMC2169625

[CR16] Hägele, C., Schlagenhauf, F., Rapp, M., Sterzer, P., Beck, A., Bermpohl, F., Stoy, M., Ströhle, A., Wittchen, H.-U., Dolan, R. J., & Heinz, A. (2015). Dimensional psychiatry: Reward dysfunction and depressive mood across psychiatric disorders. *Psychopharmacology,**232*(2), 331–341. 10.1007/s00213-014-3662-7 24973896 10.1007/s00213-014-3662-7 PMC4297301

[CR17] Halperin, I., & Vigotsky, A. D. (2024). An integrated perspective of effort and perception of effort. *Sports Medicine,**54*(8), 2019–2032. 10.1007/s40279-024-02055-8 38909350 10.1007/s40279-024-02055-8 PMC11329614

[CR18] Harris, C. R., Millman, K. J., van der Walt, S. J., Gommers, R., Virtanen, P., Cournapeau, D., ..., Oliphant, T. E. (2020). Array programming with NumPy. *Nature,**585*(7825), 357–362. 10.1038/s41586-020-2649-2 32939066 10.1038/s41586-020-2649-2 PMC7759461

[CR19] Harshaw, C. (2015). Interoceptive dysfunction: Toward an integrated framework for understanding somatic and affective disturbance in depression. *Psychological Bulletin,**141*(2), 311–363. 10.1037/a0038101 25365763 10.1037/a0038101 PMC4346391

[CR20] Hogan, P. S., Galaro, J. K., & Chib, V. S. (2019). Roles of ventromedial prefrontal cortex and anterior cingulate in subjective valuation of prospective effort. *Cerebral Cortex,**29*(10), 4277–4290. 10.1093/cercor/bhy310 30541111 10.1093/cercor/bhy310 PMC6735256

[CR21] Hu, E. J., Casamento-Moran, A., Galaro, J. K., Chan, K. L., Edden, R. A. E., Puts, N. A. J., & Chib, V. S. (2022). Sensorimotor cortex GABA moderates the relationship between physical exertion and assessments of effort. *The Journal of Neuroscience,**42*(31), 6121–6130. 10.1523/JNEUROSCI.2255-21.2022 35764380 10.1523/JNEUROSCI.2255-21.2022 PMC9351634

[CR22] Husain, M., & Roiser, J. P. (2018). Neuroscience of apathy and anhedonia: A transdiagnostic approach. *Nature Reviews Neuroscience,**19*(8), 470–484. 10.1038/s41583-018-0029-9 29946157 10.1038/s41583-018-0029-9

[CR23] Jurgelis, M., Chong, W. B., Atkins, K. J., Cooper, P. S., Coxon, J. P., & Chong, T.T.-J. (2021). Heightened effort discounting is a common feature of both apathy and fatigue. *Scientific Reports*. 10.1038/s41598-021-01287-2 34782630 10.1038/s41598-021-01287-2 PMC8593117

[CR24] Kroenke, K., Spitzer, R. L., & Williams, J. B. W. (2001). The PHQ-9. *Journal of General Internal Medicine,**16*(9), 606–613. 10.1046/j.1525-1497.2001.016009606.x 11556941 10.1046/j.1525-1497.2001.016009606.x PMC1495268

[CR25] Kurniawan, I. T., Guitart-Masip, M., Dayan, P., & Dolan, R. J. (2013). Effort and valuation in the brain: The effects of anticipation and execution. *The Journal of Neuroscience,**33*(14), 6160–6169. 10.1523/JNEUROSCI.4777-12.2013 23554497 10.1523/JNEUROSCI.4777-12.2013 PMC3639311

[CR26] Kurniawan, I. T., Seymour, B., Talmi, D., Yoshida, W., Chater, N., & Dolan, R. J. (2010). Choosing to make an effort: The role of striatum in signaling physical effort of a chosen action. *Journal of Neurophysiology,**104*(1), 313–321. 10.1152/jn.00027.2010 20463204 10.1152/jn.00027.2010 PMC2904211

[CR27] Le Heron, C., Plant, O., Manohar, S., Ang, Y.-S., Jackson, M., Lennox, G., Hu, M. T., & Husain, M. (2018). Distinct effects of apathy and dopamine on effort-based decision-making in Parkinson’s disease. *Brain,**141*(5), 1455–1469. 10.1093/brain/awy110 29672668 10.1093/brain/awy110 PMC5917786

[CR28] Lopez-Gamundi, P., Yao, Y.-W., Chong, T.T.-J., Heekeren, H. R., Mas-Herrero, E., & Marco-Pallarés, J. (2021). The neural basis of effort valuation: A meta-analysis of functional magnetic resonance imaging studies. *Neuroscience and Biobehavioral RevieWs,**131*, 1275–1287. 10.1016/j.neubiorev.2021.10.024 34710515 10.1016/j.neubiorev.2021.10.024

[CR29] Maddox, W. T., Gorlick, M. A., Worthy, D. A., & Beevers, C. G. (2012). Depressive symptoms enhance loss-minimization, but attenuate gain-maximization in history-dependent decision-making. *Cognition,**125*(1), 118–124. 10.1016/j.cognition.2012.06.011 22801054 10.1016/j.cognition.2012.06.011 PMC3426306

[CR30] Manohar, S. G., Chong, T.T.-J., Apps, M. A. J., Batla, A., Stamelou, M., ..., Husain, M. (2015). Reward pays the cost of noise reduction in motor and cognitive control. *Current Biology,**25*(13), 1707–1716. 10.1016/j.cub.2015.05.038 26096975 10.1016/j.cub.2015.05.038 PMC4557747

[CR31] Marin, R. S., Biedrzycki, R. C., & Firinciogullari, S. (1991). Reliability and validity of the apathy evaluation scale. *Psychiatry Research,**38*(2), 143–162. 10.1016/0165-1781(91)90040-V 1754629 10.1016/0165-1781(91)90040-v

[CR32] Müller, T., Klein-Flügge, M. C., Manohar, S. G., Husain, M., & Apps, M. A. J. (2021). Neural and computational mechanisms of momentary fatigue and persistence in effort-based choice. *Nature Communications,**12*(1), 1. 10.1038/s41467-021-24927-7 10.1038/s41467-021-24927-7 PMC8319292 34321478

[CR33] Norbury, A., Hauser, T. U., Fleming, S. M., Dolan, R. J., & Huys, Q. J. M. (2024). Different components of cognitive-behavioral therapy affect specific cognitive mechanisms. *Science Advances,**10*(13), Article eadk3222. 10.1126/sciadv.adk3222 38536924 10.1126/sciadv.adk3222 PMC10971416

[CR34] Pedregosa, F., Varoquaux, G., Gramfort, A., Michel, V., Thirion, B., Grisel, O., Blondel, M., Prettenhofer, P., Weiss, R., Dubourg, V., Vanderplas, J., Passos, A., Cournapeau, D., Brucher, M., Perrot, M., & Duchesnay, É. (2011). Scikit-learn: Machine learning in Python. *Journal of Machine Learning Research,**12*(85), 2825–2830.

[CR35] Peirce, J., Gray, J. R., Simpson, S., MacAskill, M., Höchenberger, R., Sogo, H., Kastman, E., & Lindeløv, J. K. (2019). PsychoPy2: Experiments in behavior made easy. *Behavior Research Methods,**51*(1), 195–203. 10.3758/s13428-018-01193-y 30734206 10.3758/s13428-018-01193-y PMC6420413

[CR36] R Core Team. (2016). *R: a language and environment for statistical computing.* [Computer software]. Vienna: R Foundation for Statistical Computing. https://www.R-project.org

[CR37] Rottenberg, J., Gross, J. J., & Gotlib, I. H. (2005). Emotion context insensitivity in major depressive disorder. *Journal of Abnormal Psychology,**114*(4), 627–639. 10.1037/0021-843X.114.4.627 16351385 10.1037/0021-843X.114.4.627

[CR38] Ruggiero, V., Dell’Acqua, C., Cremonese, E., Giraldo, M., & Patron, E. (2025). Under the surface: Low cardiac vagal tone and poor interoception in young adults with subclinical depressive symptoms. *Journal Of Affective Disorders,**375*, 1–9. 10.1016/j.jad.2025.01.057 39826615 10.1016/j.jad.2025.01.057

[CR39] Salamone, J. D., Yohn, S. E., López-Cruz, L., San Miguel, N., & Correa, M. (2016). Activational and effort-related aspects of motivation: Neural mechanisms and implications for psychopathology. *Brain,**139*(5), 1325–1347. 10.1093/brain/aww050 27189581 10.1093/brain/aww050 PMC5839596

[CR40] Schwarz, G. (1978). Estimating the dimension of a model. *The Annals of Statistics,**6*(2), 461–464.

[CR41] Smith, R., Feinstein, J. S., Kuplicki, R., Forthman, K. L., Stewart, J. L., Paulus, M. P., ..., Khalsa, S. S. (2021). Perceptual insensitivity to the modulation of interoceptive signals in depression, anxiety, and substance use disorders. *Scientific Reports,**11*(1), Article 2108. 10.1038/s41598-021-81307-3 33483527 10.1038/s41598-021-81307-3 PMC7822872

[CR42] The pandas development team. (2024). *Pandas-dev/pandas: Pandas (v2.2.2). Zenodo.* [Computer software]. 10.5281/zenodo.10957263

[CR43] Treadway, M. T., Bossaller, N. A., Shelton, R. C., & Zald, D. H. (2012). Effort-based decision-making in major depressive disorder: A translational model of motivational anhedonia. *Journal of Abnormal Psychology,**121*(3), 553–558. 10.1037/a0028813 22775583 10.1037/a0028813 PMC3730492

[CR44] Treadway, M. T., Buckholtz, J. W., Schwartzman, A. N., Lambert, W. E., & Zald, D. H. (2009). Worth the ‘EEfRT’? The effort expenditure for rewards task as an objective measure of motivation and anhedonia. *PLoS One,**4*(8), e6598. 10.1371/journal.pone.0006598 19672310 10.1371/journal.pone.0006598 PMC2720457

[CR45] Valton, V., Mkrtchian, A., Moses-Payne, M., Gray, A., Kieslich, K., VanUrk, S., ..., Roiser, J. P. (2024). *A computational approach to understanding effort-based decision-making in depression*. 10.1101/2024.06.17.599286 10.1017/S0033291725101967 PMC12527505 41059633

[CR46] Vinckier, F., Jaffre, C., Gauthier, C., Smajda, S., Abdel-Ahad, P., Le Bouc, R., Daunizeau, J., Fefeu, M., Borderies, N., Plaze, M., Gaillard, R., & Pessiglione, M. (2022). Elevated effort cost identified by computational modeling as a distinctive feature explaining multiple behaviors in patients with depression. *Biological Psychiatry: Cognitive Neuroscience and Neuroimaging,**7*(11), 1158–1169. 10.1016/j.bpsc.2022.07.011 35952972 10.1016/j.bpsc.2022.07.011

[CR47] Virtanen, P., Gommers, R., Oliphant, T. E., Haberland, M., Reddy, T., Cournapeau, D., Burovski, E., Peterson, P., Weckesser, W., Bright, J., van der Walt, S. J., Brett, M., Wilson, J., Millman, K. J., Mayorov, N., Nelson, A. R. J., Jones, E., Kern, R., Larson, E., … van Mulbregt, P. (2020). SciPy 1.0: Fundamental algorithms for scientific computing in Python. *Nature Methods,**17*(3), 261–272. 10.1038/s41592-019-0686-2 32015543 10.1038/s41592-019-0686-2 PMC7056644

[CR48] Weilharter, F., Rewitz, K., Halperin, I., & Wolff, W. (2024). The relationship between prescribed ratings of perceived exertion and force production in repeated isometric contractions. *Psychology of Sport and Exercise,**73*, Article 102657. 10.1016/j.psychsport.2024.102657 38719021 10.1016/j.psychsport.2024.102657

[CR49] Wickham, H. (with Sievert, C.). (2016). *ggplot2: Elegant graphics for data analysis* (Second edition). Springer international publishing.

[CR50] Wolpe, N., Aymerich, C., Jin, Y., Martin-Subero, M., Fuentes-Perez, P., Ovejas-Catalan, C., ..., Fernandez-Egea, E. (2025). *Characterising Negative Symptoms in Schizophrenia: CHANSS study protocol* (p. 2025.05.30.25328412). medRxiv. 10.1101/2025.05.30.25328412 10.1192/bjo.2025.10880 PMC12641429 41195453

[CR51] Wolpe, N., Holton, R., & Fletcher, P. C. (2024). What is mental effort: A clinical perspective. *Biological Psychiatry,**95*(11), 1030–1037. 10.1016/j.biopsych.2024.01.022 38309319 10.1016/j.biopsych.2024.01.022

[CR52] Xiao, B., & Wolpe, N. (2025). The value of progress feedback in physical effort-based decision making. *Motivation Science*. 10.1037/mot0000411

